# Protein engineering strategies for microbial production of isoprenoids

**DOI:** 10.1016/j.mec.2020.e00129

**Published:** 2020-05-16

**Authors:** Georgios Daletos, Gregory Stephanopoulos

**Affiliations:** Department of Chemical Engineering, Massachusetts Institute of Technology, 77 Massachusetts Avenue, Cambridge, MA 02139, United States

**Keywords:** Enzyme engineering, Activity, Selectivity, Promiscuity, Isoprenoids, Microbial hosts

## Abstract

Isoprenoids comprise one of the most chemically diverse family of natural products with high commercial interest. The structural diversity of isoprenoids is mainly due to the modular activity of three distinct classes of enzymes, including prenyl diphosphate synthases, terpene synthases, and cytochrome P450s. The heterologous expression of these enzymes in microbial systems is suggested to be a promising sustainable way for the production of isoprenoids. Several limitations are associated with native enzymes, such as low stability, activity, and expression profiles. To address these challenges, protein engineering has been applied to improve the catalytic activity, selectivity, and substrate turnover of enzymes. In addition, the natural promiscuity and modular fashion of isoprenoid enzymes render them excellent targets for combinatorial studies and the production of new-to-nature metabolites. In this review, we discuss key individual and multienzyme level strategies for the successful implementation of enzyme engineering towards efficient microbial production of high-value isoprenoids. Challenges and future directions of protein engineering as a complementary strategy to metabolic engineering are likewise outlined.

## Highlights

• Isoprenoid enzymes are attractive biocatalysts for protein engineering.
• Isoprenoid enzymes can be engineered for broader substrate promiscuity.
• Protein engineering can lead to the production of non-natural isoprenoids.
• Protein engineering can promote co-localization of isoprenoid pathway enzymes.
• Protein engineering supplements combinatorial biosynthesis for isoprenoid synthesis.

## 1 Introduction

**Fig. 1:**
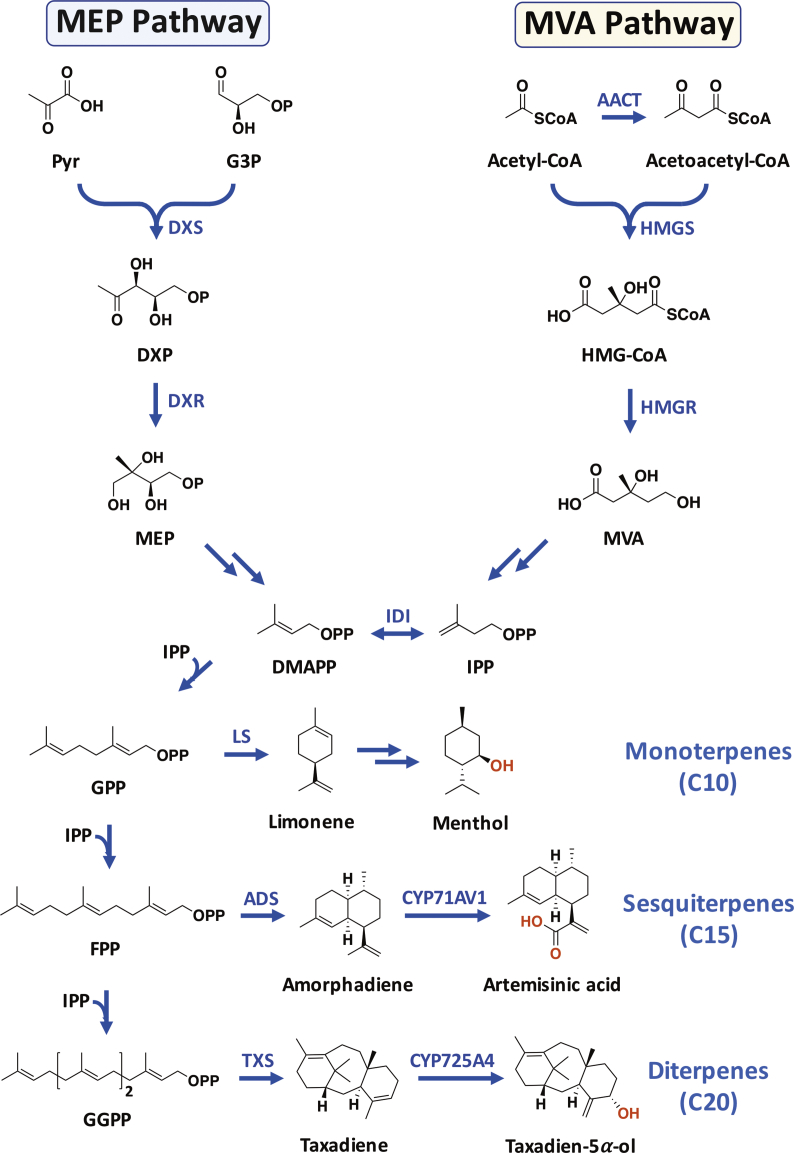
Schematic representation of isoprenoid biosynthesis. Key biosynthetic steps in the mevalonate (MVA) and 2-C-methyl-d-erythritol-4-phosphate (MEP) pathways, along with selected examples of monoterpenes, sesquiterpenes, and diterpenes, are illustrated. Double arrows indicate multiple enzymatic steps. Abbreviations: AACT, acetoacetyl-coenzyme A thiolase; Pyr, pyruvate; G3P, glyceraldehyde 3-phosphate; DXS, 1-deoxy-d-xylulose-5-phosphate synthase; DXR, 1-deoxy-d-xylulose-5-phosphate reductoisomerase; HMGS, 3-hydroxy-3-methylglutaryl coenzyme A synthase; HMGR, 3-hydroxy-3-methylglutaryl coenzyme A reductase; IDI, isopentenyl diphosphate isomerase; GPP, geranyl diphosphate; FPP, farnesyl diphosphate; GGPP, geranylgeranyl diphosphate; LS, limonene synthase; ADS, amorpha-4,11-diene synthase; TXS, taxa-4(5),11(12)-diene synthase; CYP71AV1, amorpha-4,11-diene 12-hydroxylase; CYP725A4, taxa-4(5),11(12)-diene 5*α*-hydroxylase.

Isoprenoids (also known as terpenoids) comprise one of the most diverse and important classes of natural products with a broad spectrum of biological activities. Biosynthetically, isoprenoids are derived from two distinct metabolic pathways, the 2-C-methyl-d-erythritol-4-phosphate (MEP) pathway, which is present in most bacteria and plastids of plant cells, and the mevalonate (MVA) pathway, which functions in eukaryotes, archaea, and certain bacteria (“upstream” pathways of isoprenoids). Both routes lead to the formation of the five-carbon constitutional isomers isopentenyl diphosphate (IPP) and dimethylallyl diphosphate (DMAPP), the building blocks of isoprenoids. Subsequently, the basic isoprene units IPP and DMAPP are condensed in an incremental manner by prenyl diphosphate synthases leading to the formation of various size linear prenyl chains, such as geranyl diphosphate (GPP), farnesyl diphosphate (FPP), and geranylgeranyl diphosphate (GGPP), the isoprenoid precursors of monoterpenes, sesquiterpenes, and diterpenes, respectively (Fig. 1).

Enzymes belonging to the family of terpene synthases catalyze the conversion steps of the resulting isoprenoid chains into the core scaffold of isoprenoids. The derivatization reactions are initiated through multistep bond-forming carbocations and hydride, methyl, or methylene migrations (Greenhagen et al., 2006). The derivatization is terminated by elimination or aqueous quenching of carbocation intermediates to afford a vast array of hydrocarbon and oxygenated terpene scaffolds (Lauchli et al., 2013). These reactions are catalyzed by class I and II terpene cyclases, which employ different mechanisms to ensure the formation of carbocation intermediates. Interestingly, more complex terpenoid synthase structures have been discovered containing both class I and II domains (e.g., abietadiene synthase), leading to high structural diversity (Köksal et al., 2011).

Finally, the basic terpenoid skeleton of terpenes is further diversified in a regio- and stereoselective manner through a series of post-modifications. These modifications commonly start with oxidation catalyzed by heme-containing enzymes, namely cytochrome P450 monooxygenases (CYP450s, CYPs), which introduce one atom of molecular oxygen into nonactivated C–H bonds (Urlacher and Girhard, 2019). Apart from the incorporation of oxygen atoms into the substrates, these enzymes also catalyze selective dealkylation, reduction, deamination, C–C bond formation and cleavage, among other reaction types (Renault et al., 2014). Following oxidation, other functional groups, such as acyl-, aryl-, or sugar moieties, may be added. These chemical modifications contribute to the diversification of isoprenoids and are essential for their biological functionality (Janocha et al., 2015). Importantly, both terpene synthases and cytochrome P450s control the regio- and stereochemistry of produced isoprenoids, which is a challenging synthetic task via conventional chemical routes (Andersen-Ranberg et al., 2016; Xiao et al., 2019).

The main enzymes involved in isoprenoid biosynthesis, including prenyl diphosphate synthases, terpene synthases, and cytochrome P450s, are considered attractive biocatalysts for biotechnological applications, due to their diverse spectrum of catalytic activities on a wide range of complex molecules. However, the performance of wild-type enzymes in heterologous microbial systems does not always match the process requirements, owing to low catalytic efficiency or limited stability. In these cases, protein engineering is a prerequisite to improving the performance of enzymes beyond their natural capabilities. In this review, we highlight key protein engineering strategies involving the aforementioned classes of enzymes that have been successfully applied for enhancing the production of value-added isoprenoids in microbial hosts. We also discuss pitfalls associated with these engineering strategies and provide solutions on how these limitations could be overcome in this fascinating and highly evolving field.

## 2 Individual enzyme engineering strategies

### 2.1 Altering enzyme catalytic activity

**Fig. 2:**
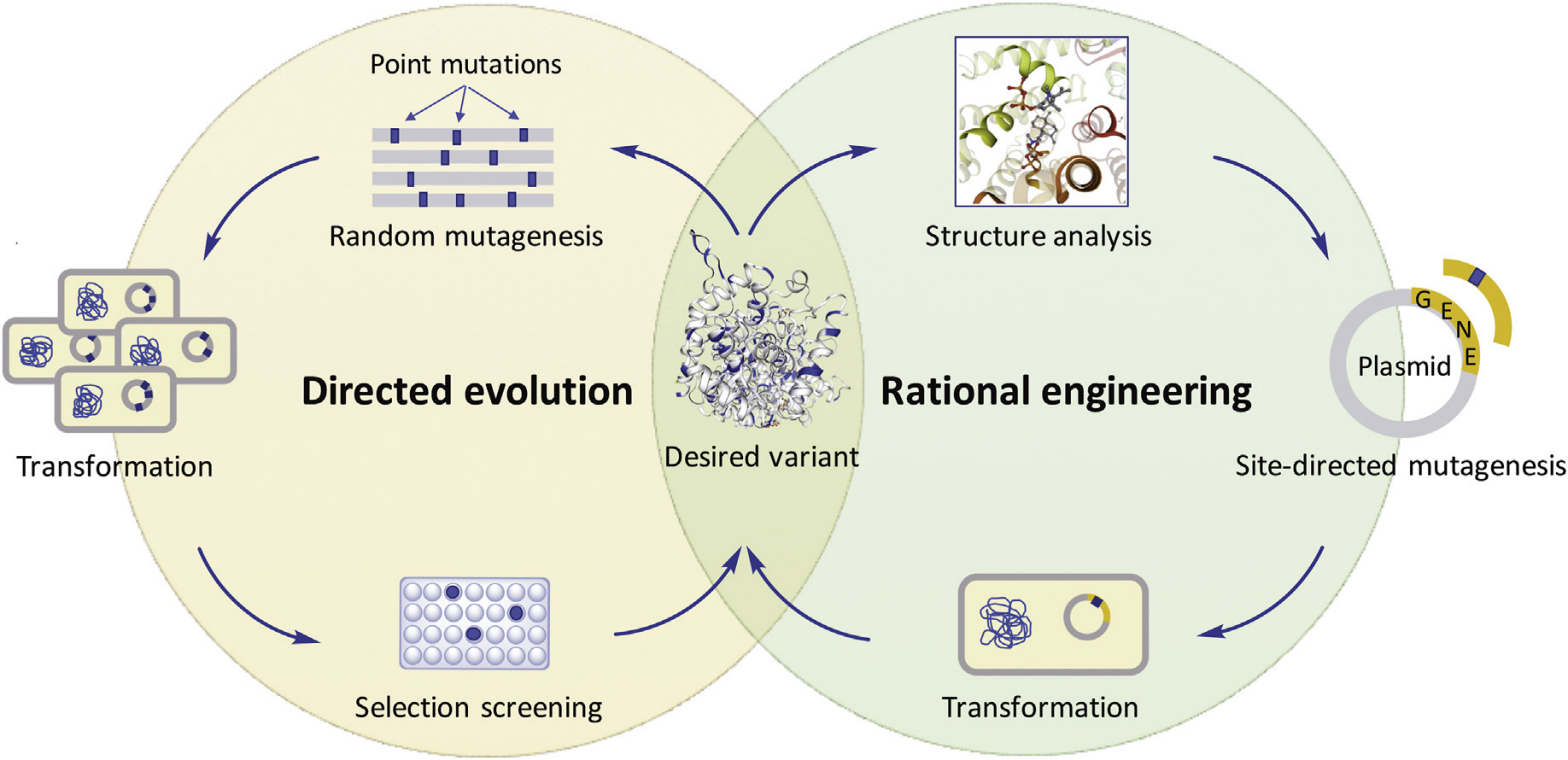
Schematic illustration of protein engineering strategies. Directed evolution and rational engineering are complementary approaches and their combination is commonly the preferred option for optimal construction of the desired enzyme variants.

**Fig. 3:**
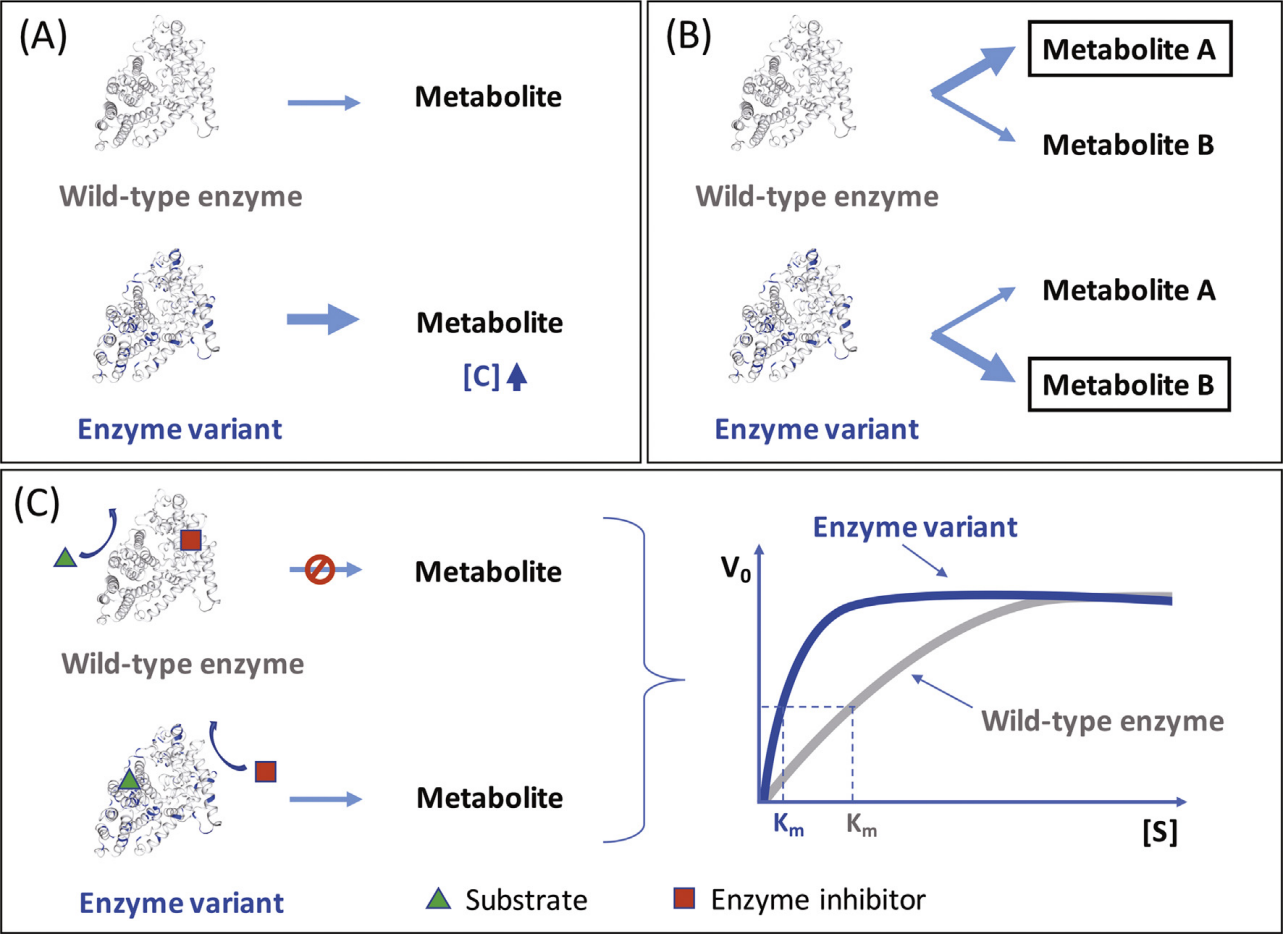
Engineering approaches of individual enzymes for increased production of isoprenoids. (A) Enhancement of catalytic activity, (B) Altering catalytic specificity, and (C) Deregulation of feedback inhibition. For simplicity reasons, the hypothetical enzyme follows Michaelis-Menten kinetics. Enzyme structural models were constructed using the SWISS-MODEL server (Bienert et al., 2016).

Heterologous expression of isoprenoid enzymes in microbial host systems has met with great success in the last few years. However, wild-type enzymes typically show low catalytic activity or stability. Engineering of proteins for improved performance is of major interest, as supported by numerous studies appearing in the literature (Styles et al., 2017; Xiao et al., 2019). The most successful strategies to obtain improved variants of enzymes are directed evolution and rational engineering (Fig. 2). The former approach mimics natural selection. It involves the generation of a library of mutated enzymes employing iterative cycles of random mutagenesis techniques (e.g., error-prone polymerase chain reaction) or recombination technology (e.g., gene shuffling) followed by screening until the desired variant is obtained (Steiner and Schwab, 2012). On the other hand, rational engineering requires a solid knowledge of the role of key amino acid residues that are involved in the catalytic activity or specificity of an enzyme. These data are commonly obtained from the analysis of existing protein structures or homology models. In this regard, site-directed mutagenesis is usually employed to rationally improve the performance of an enzyme (Greenhagen et al., 2006). In contrast to rational engineering, directed evolution has the advantage that no structural or mechanistic information of proteins is required, and thus it can be performed on any given enzyme. However, this increases the number of variants that need to be screened, which involves the development of appropriate high-throughput assays (Diaz et al., 2011). The combination of rational protein design and directed evolution (semi-rational approach) is an alternative way to obtain improved characteristics of enzymes by combining the advantages of these two strategies (Fig. 2, Fig. 3A) (Bloom et al., 2005; Steiner and Schwab, 2012).

Ginkgolides, the active constituents of *Ginkgo biloba* extract, have been the subject of extensive studies due to their remarkable anti-inflammatory, anti-apoptotic, and neuroprotective effects (Gu et al., 2012; Zhou et al., 2016). The gateway precursor of ginkgolides is the diterpenoid levopimaradiene, which is derived from GGPP through catalysis by levopimaradiene synthase (LPS). In an attempt to increase levopimaradiene production, Leonard et al. (2010) constructed a homology structure for the active site of LPS as a guide to probe essential residues for catalytic function. Phylogeny-based mutation showed that the residues M593 and Y700 were significant for the production phenotype. The LPS evolvability profile was obtained through saturation mutagenesis of these residues, indicating that the M593I and Y700F variants elevated productivity up to 5-fold. The combination of these individual mutations proved to be advantageous, resulting in a 10-fold increase in the levopimaradiene titer (Leonard et al., 2010). In the case of geranylgeranyl diphosphate synthase (GGPPS), due to the lack of a suitable structural model, the lycopene biosynthetic pathway was utilized as a colorimetric reporter. Improved GGPPS variants were identified by more intense red coloration than the wild-type GGPPS as a result of increased production of lycopene. The combination of the respective high-producing GGPPS and LPS mutants, along with directing the flux toward IPP and DMAPP precursors through metabolic engineering, resulted in a 2600-fold increase over the pathway harboring wild-type GGPPS and LPS alone, overcoming the GGPPS-LPS limited inherent capacity (Leonard et al., 2010).

Another promising approach for increasing the catalytic activity of enzymes is cofactor engineering. Cofactors are considered essential elements for the activity of isoprenoid enzymes. Sesquiterpene synthases have been reported to accept the divalent cation ion Mg^2+^ as a cofactor, whereas conifer monoterpene synthases, such as pinene synthase, are selective to Mn^2+^. However, the concentration of Mn^2+^ in the cytosol of *Escherichia coli* (ca. 10 ​μM) is significantly lower than the concentration of Mg^2+^(10–20 ​mM), thus posing a limiting factor for the production of pinene. Interestingly, following one round of mutagenesis and screening, the metal dependency of the resulting pinene synthase variant shifted from Mn^2+^ to Mg^2+^, thus enabling enhanced pinene productivity (140 mg/L/24 h in flask culture) in *E. coli* (Tashiro et al., 2016).

Inactivation – rather than an enhancement – of the catalytic activity of unspecific domains of multifunctional enzymes, may lead to enhanced production of the desired metabolite. This is exemplified in the case of the bifunctional lycopene cyclase/phytoene synthase (CrtYB) from *Xanthophyllomyces dendrorhous*, which catalyzes the synthesis of phytoene and *β*-carotene, respectively (Xie et al., 2015). In particular, disruption of the lycopene cyclase functional domain of CrtYB through directed evolution resulted in various enzyme variants that only retained the phytoene synthase function. As a result, expression of the respective variants in *Saccharomyces cerevisiae* led to a 3-fold enhancement (2.53 ​mg/g dry cell weight) in lycopene production compared to an *S. cerevisiae* strain overexpressing the enzyme phytoene synthase (CrtB) from *Pantoea agglomerans* (Xie et al., 2015).

*In vivo* properties, such as solubility and stability, are often the limiting factors for the activity profiles of enzymes in microbes (Styles et al., 2017; Yoshikuni et al., 2008). This holds especially true in the case of non-native proteins (e.g., plant-derived isoprenoid enzymes) that are expressed in heterologous systems. Codon-optimization and promoter engineering are commonly employed to increase the expression level of heterologous proteins (Elena et al., 2014; Gustafsson et al., 2004). However, further improvement of *in vivo* enzymatic properties might prove beneficial for optimal function and flux of synthetic metabolic pathways. For example, despite the fact that the humulene synthase (HUM) and the N-terminal truncated, deregulated form of 3-hydroxyl-3-methylglutaryl-CoA reductase (tHMGR; see 2.3. Deregulation of feedback inhibition for further details) were highly expressed, neither enzyme showed sufficient activity, indicating that they are the primary bottlenecks in humulene production (Yoshikuni et al., 2008). By conservation analysis of over 200 different *E. coli* enzymes, glycine (Gly) and proline (Pro) were identified as favorable residues for enhancing the *in vivo* properties thereof. Accordingly, these amino acids were selectively introduced to tHMGR and HUM on the basis of their evolutionary relationships and predicted profiles. The effect of these mutations on the *in vivo* properties of tHMGR and HUM variants was found to be cumulative without essentially impacting the respective catalytic activity and product specificity compared to the wild-type HUM. As such, the humulene productivity of *E. coli* harboring the tHMGR and HUM mutants was increased by around 1000-fold (Yoshikuni et al., 2008). Further investigation of the contribution of Gly and Pro-mediated improvement in sesquiterpene production, showed that these residues increased the ability of mutant variants to fold properly at higher temperature conditions. As a result, sesquiterpene production was enhanced from 3.3-fold (at 20 ​°C) to 220-fold (at 37 ​°C), suggesting that enzyme stability in heterologous systems is a promising strategy for eliminating the bottleneck of inefficient enzymes.

C-terminal truncation of terpene synthases has likewise been found to increase thermostability without negatively affecting their kinetics or product profiles (Styles et al., 2017). The C-terminal tail is suggested to play an important role in protein localization and protein-protein interaction, supporting the fact that there is no loss in catalytic efficiency. Similarly, membrane-bound plant cytochromes P450 pose great challenges during heterologous pathway engineering in microbial hosts due to low solubility and expression levels (Renault et al., 2014). To avoid these pitfalls, truncation of the N-terminal membrane anchor region is typically required. In addition, studies have shown that the replacement of the N-terminal membrane sequence by solubilizing leader peptides or other anchor regions from highly-expressed heterologous P450s promotes the catalytic performance of enzymes (Xiao et al., 2019). For example, replacement of the N-terminal membrane anchor region of CYP76AH4 from *Rosmarinus officinalis* by the lysine- and serine-rich leader sequence MAKKTSSKGK resulted in the functional expression of CYP76AH4 in *E. coli* catalyzing the oxidation of abietatriene to the diterpenoid ferruginol (Zi and Peters, 2013). Likewise, the N-terminal sequence of the bovine microsomal 17*α*-hydroxylase cytochrome P450 (P45017*α*) led to a 5-fold improvement in the productivity of 8-cadinene hydroxylase (105 ​mg/L), which catalyzes the conversion of cadinene to 8-hydroxycadinene (Barnes et al., 1991; Chang et al., 2007).

### 2.2 Altering enzyme specificity

Catalytic promiscuity of enzymes with regard to their substrate range is believed to be an important determinant in molecular evolution allowing proteins to acquire higher specificity or novel functions in response to environmental changes (Yoshikuni et al., 2006). Additionally, several studies have indicated that terpene synthases show a drastic shift in their activities with a surprisingly small number of specific amino acid substitutions, exhibiting high catalytic plasticity (Hammer et al., 2013). As such, isoprenoid pathway enzymes are excellent models for promiscuity engineering studies (Fig. 3B) (Hammer et al., 2017; Pazouki and Niinemets, 2016). For example, the highly promiscuous enzyme *γ*-humulene synthase from *Abies grandis* was shown to produce more than fifty different sesquiterpenes solely from FPP as a substrate. Yoshikuni et al. (2006) identified nineteen active-site residues contributing to the plasticity of *γ*-humulene synthase based on a homology structure of the later using the crystal structure of 5-*epi*-aristolochene synthase as a model. As a result, saturation mutagenesis of selected residues W315, M447, S484 and Y566 at the active site, significantly shifted sesquiterpene selectivity up to 1000-fold. Moreover, systematic recombination of the identified residues based on a mathematical model indicated that most of the mutations introduced into the enzyme play a shared role in the catalytic outcome (Yoshikuni et al., 2006).

Another example of a promiscuous enzyme is taxadiene synthase, which produces the Taxol precursor taxa-4(5)-11(12)-diene and the alternative cyclization product taxa-4(20)-11(12)-diene at 92% and 8%, respectively (Edgar et al., 2017) (Fig. 1). Hydroxylation of the former compound by taxadiene-5*α*-hydroxylase (CYP725A4) affords an epoxide intermediate which undergoes nonspecific degradation to a range of byproducts when expressed in *E. coli*. On the other hand, the minor product taxa-4(20)-11(12)-diene was shown to be hydroxylated with high specificity by CYP725A4 directing the flux toward the production of the desired Taxol precursor taxadien-5*α*-ol. As such, taxadiene synthase was engineered to improve selectivity for taxa-4(20)-11(12)-diene, alleviating the early paclitaxel biosynthetic pathway bottleneck (Edgar et al., 2017).

Cytochromes P450 are a class of highly promiscuous enzymes acting on diverse substrates, including alkanes, fatty acids, terpeneoids, vitamins, and steroids, among others (Guo et al., 2016; Urlacher and Girhard, 2019). Prominent examples are the well characterized enzymes P450_BM3_ from *Bacillus megaterium* and the P450_cam_ from *Pseudomonas putida*. These enzymes possess many advantageous properties, such as increased solubility and stability, fast reaction rates, and high-yield expression in bacteria (Janocha et al., 2015; Xiao et al., 2019). The natural substrates of P450_BM3_ and P450_cam_ include long-chain saturated fatty acids and D-(+)-camphor, respectively (Sowden et al., 2005). Nevertheless, these enzymes catalyze oxidation reactions of non-native substrates due to catalytic promiscuity. Moreover, they have demonstrated high re-engineering capacity, which renders them ideal targets for protein engineering studies and *de novo* pathway design of complex isoprenoids in microbial hosts (Sowden et al., 2005). A notable example of successful utilization of P450s is the microbial production of the antimalarial drug artemisinin. The native enzyme from *Artemisia annua* (CYP71AV1) catalyzes the conversion of amorpha-4,11-diene to artemisinic acid (Fig. 1). However, this enzyme is not functionally expressed in *E. coli* leading to low product yield. To overcome this limitation, the non-native enzyme P450_BM3_ was employed for protein engineering studies. Saturation mutagenesis of key residues in the active site of P450_BM3_ based on transition state complex modeling with amorphadiene, led to a mutant that was able to perform epoxidation of amorpha-4,11-diene through a novel route. Interestingly, the resulting artemisinic epoxide could be transformed to dihydroartemisinic acid by high-yielding synthetic chemistry, overcoming the regioselective reduction bottleneck of the native metabolite artemisinic acid (Dietrich et al., 2009). In addition, the novel semi-biosynthetic route was found to be optimal at 30 ​°C and reduced the culture time from seven to two days. Thus, protein engineering could be exploited for the rational design of enzymes with broader substrate promiscuity, opening new perspectives to access complex isoprenoids, whose biosynthesis is limited by the low catalytic rates of native enzymes.

Monoterpenes have been valuable compounds as food and cosmetic additives and have recently received increased attention as pharmaceutical agents, pesticides, and advanced biofuels (Vespermann et al., 2017; Zhuang et al., 2019). However, the efficiency of monoterpene production in host cells is limited by the low levels of the GPP pool. Hence, the overproduction of GPP is considered an effective strategy for efficient microbial synthesis of monoterpenes. In yeast, the enzyme Erg20p catalyzes the stepwise synthesis of GPP and FPP via sequential coupling of IPP with DMAPP and GPP, respectively. It is suggested that the sequential activity of Erg20p is a limiting factor for the production of monoterpenes, due to the conversion of GPP to FPP. In addition, Erg20p knockout mutants are unable to synthesize ergosterol, an essential component of the cell membranes (Ignea et al., 2014). To this end, it is necessary to engineer Erg20p into a specific geranyl diphosphate synthase (GPPS) while reducing the production of FPP to the minimum for adequate sterol biosynthesis (Stanley Fernandez et al., 2000). Through modeling experiments of Erg20p, Ignea et al. (2014) found mutations that hindered the FPP synthase activity of Erg20p without affecting the synthesis of GPP, thus improving sabinene production by 10-fold (0.53 ​mg/L) (Ignea et al., 2014).

Recombination of related sequences from structurally similar proteins (chimeragenesis), has been a promising approach for engineering product specificity in terpene synthases (Li et al., 2013). The advantage of chimeragenesis is that it merely uses phylogenetic information without the need for defining the minimum structural requirements for catalytic specificity (Greenhagen et al., 2006). However, this approach is limited to proteins with higher than 70% sequence homology (Bloom et al., 2005). Interestingly, domain-swapping experiments between two evolutionarily related classes of enzymes, namely *Nicotiana tabacum* 5-*epi*-aristolochene synthase (TEAS) and *Hyoscyamus muticus* premnaspirodiene synthase (HPS), showed that phylogenetic variation in amino acids residing outside the active site play an important role in the reaction product specificity of these terpene synthases (Greenhagen et al., 2006). Thus, apart from specific residues located at the active site, distal residues may also be of high importance for the catalytic outcome, probably by modulating the geometry and dynamics of the active site. These results highlight the potential of chimeragenesis as a powerful tool for predicting beneficial mutations at unexpected sites of enzymes (Bloom et al., 2005; Greenhagen et al., 2006).

The possibilities of enzyme promiscuity applications in protein engineering are manifold and apart from broadening the substrate range, enzymes could be engineered for the production of non-natural isoprenoids with a diverse set of novel properties and bioactivities (Hammer et al., 2017; Li et al., 2019). For example, novel 11-carbon (C11) terpene scaffolds were produced by introducing a GPP methyltransferase, which methylates the monoterpene precursor GPP affording 2-methyl-GPP (2meGPP) (Ignea et al., 2018). The utilization efficiency of 2meGPP by different monoterpene synthases mounted up to 25%, suggesting that it is possible to increase the terpene chemical space by using non-native substrates. Due to the fact that 2meGPP is bulkier than GPP, the sites N338 and I451 at the bottom of the active site cavity of 1,8-cineole synthase from *Salvia fruticosa* (*Sf*CinS1) were selected for mutagenesis, facilitating the additional space required in the active site. Accordingly, the double variant *Sf*CinS1(N388S–I451A) showed substantial improvement in yield and increased C11 product specificity, affording almost exclusively 2-methylmyrcene. In another set of experiments, substitution of phenylalanine by tyrosine at position 571 in *Sf*CinS1 resulted in a 6-fold improvement in the affinity for 2meGPP without compromising the overall catalytic efficiency, further increasing the ratio toward C11 terpenes in yeast. The single-residue switch approach was likewise applied to other monoterpene synthases leading to several dedicated C11 synthases (Ignea et al., 2018).

The enzyme-substrate docking strategy has been successfully employed in the case of prenyl diphosphate synthases, which have been engineered to accept longer or shorter substrates by removal or addition of bulky residues in the catalytic center. As an example, by replacing the phenylalanine residues at positions 112 and 113 with amino acids bearing smaller side chains in farnesyl diphosphate synthase (FPPS), variants were produced that selectively synthesize longer chain length prenyl diphosphates, such as GGPP and geranylfarnesyl diphosphate (GFPP), respectively (Tarshis et al., 1996). Similarly, Ignea et al. (2015b) engineered Erg20p into a specific GGPP synthase by substituting phenylalanine with cysteine at position 96. Co-expression of the Erg20p variant with 8-hydroxy copalyl diphosphate synthase from *Cistus creticus* resulted in a more than 70-fold improvement in sclareol production (15.4 mg/L) in shake-flask culture (Ignea et al., 2015b). These results show that canonical prenyltransferases can be engineered to become specific for unusual substrates providing a series of new-to-nature isoprenoids. This concept could be applied to other promiscuous tailoring enzymes, such as cytochromes P450, to expand the diversity of natural biosynthetic pathways (see 3.2. Combinatorial biosynthesis of isoprenoids).

### 2.3 Deregulation of feedback inhibition

Artificial metabolic pathways in heterologous hosts often suffer from low product titers and yields due to the accumulation of toxic metabolic intermediates inducing stress responses to the cell (Pröschel et al., 2015). In addition, the resulting intermediates may be consumed by competitive metabolic pathways or act as negative regulators by blocking key pathway enzymes (feedback inhibition), leading to flux imbalances (Albertsen et al., 2011). In metabolic engineering studies, several synthetic regulatory systems have been constructed for improving the expression levels of rate-limiting enzymes (Dahl et al., 2013). Deregulation of feedback inhibition via protein engineering is a successful alternative approach for modulating the overall turnover rate of intermediates to the desired product (Fig. 3C). Deoxyxylulose 5-phosphate synthase (DXS) is a regulatory enzyme in the MEP pathway, which utilizes thiamine diphosphate (ThDP) as a cofactor for the condensation reaction between pyruvate and glyceraldehyde 3-phosphate to yield 1-deoxy-d-xylulose-5-phosphate (DXP), a precursor of the isoprenoid building blocks DMAPP and IPP (Fig. 1). It has been shown that IPP/DMAPP inhibits DXS by competing with ThDP, which is a significant metabolic burden on the MEP pathway. Banerjee et al. (2016) successfully managed to alleviate the feedback inhibition of recombinant *Populus trichocarpa* DXS (*Pt*DXS) via enzyme engineering of the active site. Although the *Pt*DXS activity was slightly decreased compared to the wild-type enzyme, this study provided proof-of-concept for the partial relief of the feedback inhibition from IPP on DXS activity (Banerjee et al., 2016).

HMGR is a key enzyme in the MVA pathway, which produces mevalonic acid from HMG-CoA (Fig. 1), a rate-limiting step in sterol biosynthesis due to feedback regulation. A strategy to overcome this bottleneck is via expression of a truncated HMGR that lacks the N-terminal membrane-binding domain, which is involved in the post-translational regulation of the protein (Polakowski et al., 1998). In addition, studies have shown that the endoplasmic reticulum membrane-bound HMGR isozyme Hmg2p in *S. cerevisiae* undergoes rapid degradation in response to enhanced levels of MVA pathway products (Gardner and Hampton, 1999). However, when lysine at position 6 was substituted by arginine in the N-terminal transmembrane domain, the Hmg2p variant was found to be resistant to degradation. This is corroborated by the fact that the expression of the Hmg2p (K6R) variant, along with isopentenyl-diphosphate Delta-isomerase (IDI1) in *S. cerevisiae*, showed a 24-fold increase in the production of cineole (200 ​mg/L) compared to the reference strain (Ignea et al., 2011). Similarly, the stable Hmg2p variant led to a 20-fold increase in the squalene content (18.5 ​mg/g dry cell weight) over the parental *S. cerevisiae* strain (Mantzouridou and Tsimidou, 2010). The experimental observations from these studies indicate that deregulation of feedback inhibition via protein engineering is a promising strategy for directing the flux of highly-regulated intermediates toward the production of the desired metabolites.

## 3 Multienzyme engineering strategies

### 3.1 Enzyme co-localization

Microbial metabolite production is often limited by the inability of the heterologous enzymes to assemble in complexes or collaborate with native enzymes (Albertsen et al., 2011). This may cause loss of product as intermediates diffuse, degrade, or are utilized by competitive pathways. As a result, toxic and reactive intermediates may be released, which are detrimental to the survival of host cells (Keasling, 2010; Lee et al., 2012). A straightforward strategy for the prevention of intermediate loss is the spatial coordination of consecutive enzymes in a metabolic pathway. The close proximity of the active centers of sequential pathway enzymes ensures a high local concentration of intermediates in the vicinity of the subsequent enzyme that catalyzes the next step in the reaction, and thus it is considered an attractive way to control the flow of metabolites through a pathway while preventing their accumulation to toxic levels (Albertsen et al., 2011). This approach is inspired by natural enzymatic systems, in which sequentially acting enzymes are located in a single organelle and exhibit direct substrate channeling through a protein tunnel (e.g., tryptophan synthase) or are physically organized into functional multiprotein complexes (e.g., cellulosome complex of *Clostridium thermocellum*), thereby enhancing metabolic fluxes to downstream products (Dueber et al., 2009; Pröschel et al., 2015 ).

In this regard, enzyme fusion is a straightforward strategy, in which key pathway enzymes are expressed as a single polypeptide, bringing active sites in closer proximity for enhanced substrate channeling (Ohto et al., 2010). Sarria et al. (2014) combinatorially screened fusions between GPPS and pinene synthase, leading to a significant increase in pinene production (up to 32 ​mg/L) in *E. coli*. Interestingly, the isomer profile of *α*- and *β*-pinene was found to vary according to whether the enzymes were co-expressed or in a fusion. Moreover, the fusion of PS to GPPS at the N-terminus had a negative effect on pinene production compared with fusion at the C-terminus, indicating that the domain order plays an important role in the expression and stability of the constructed proteins (Sarria et al., 2014). Similarly, a 100-fold enhancement of the *β*-phellandrene production (3.2 mg/g dry cell weight) was achieved by constructing a fusion of *β*-phellandrene synthase to the highly expressed endogenous *cpcB* gene, encoding the *β*-subunit of phycocyanin in *Synechocystis* transformants (Formighieri and Melis, 2015). A subsequent study investigated fusions of the GPPS with the kanamycin resistance (*nptI*) and chloramphenicol resistance (*cmR*) genes as leader sequences, which are overexpressed in *Synechocystis* (Betterle and Melis, 2018). The fusion constructs enhanced the expression level of GPPS, indicating that apart from spatial confinement, highly expressed homologous (i.e., *cpcB*) or heterologous (i.e., *nptI* and *cmR*) genes can serve as leader sequences for driving higher expression of the desired gene, which is important in the case of poorly expressed isoprenoid pathway genes.

**Fig. 4:**
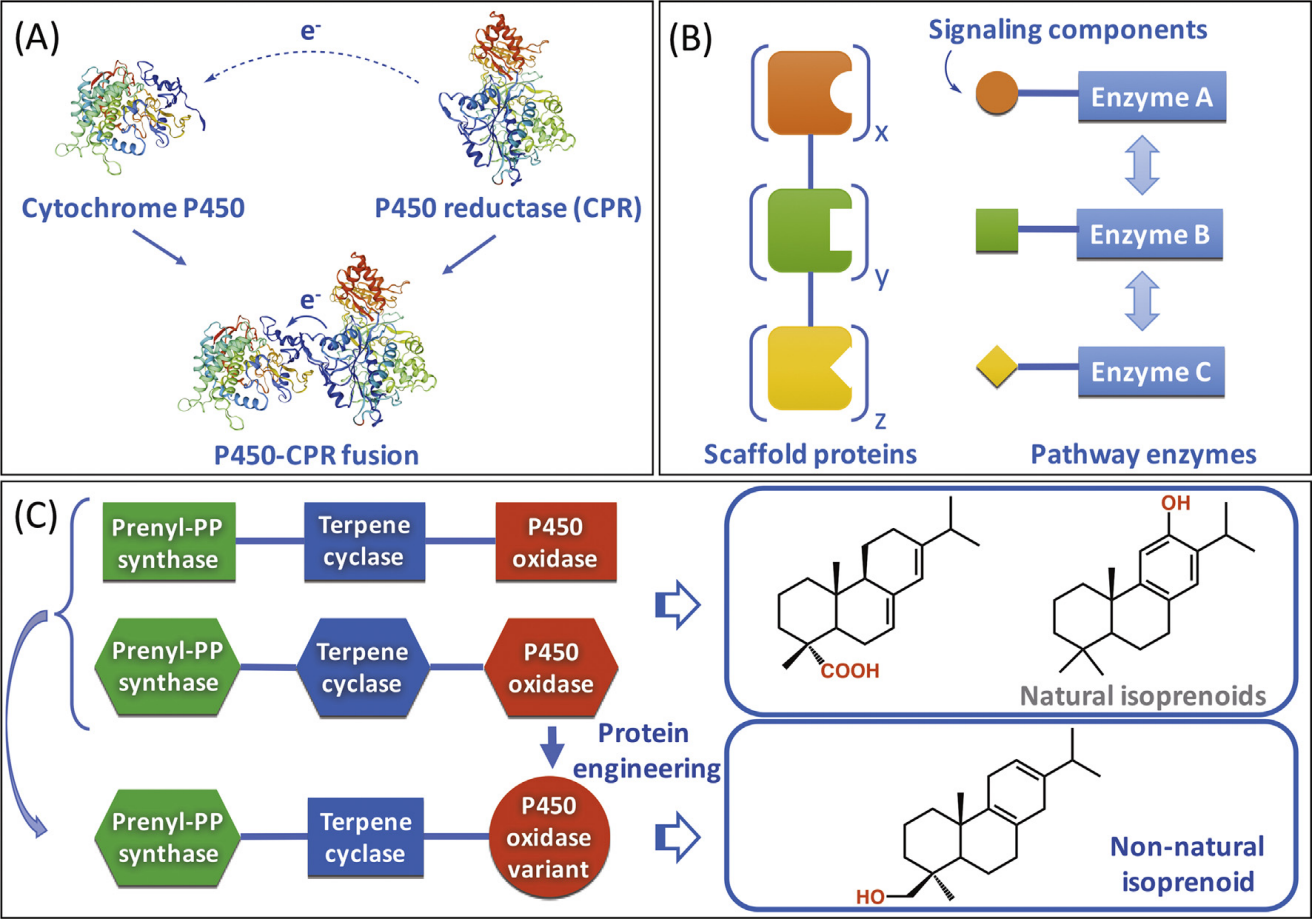
Engineering approaches of pathway enzymes for enhanced production of isoprenoids. (A) Fusion of enzymes, (B) Protein scaffolding, and (C) Construction of *de novo* pathways through combinatorial biosynthesis and protein engineering for the production of non-natural isoprenoids. Examples of natural and non-natural isoprenoids were extracted from (Guo et al., 2013; Ignea et al., 2015a; Ro et al., 2005). Enzyme structural models were constructed using the SWISS-MODEL server (Bienert et al., 2016).

P450s normally interact with electron transfer proteins/redox partners (i.e., cytochrome P450 reductases; CPRs) to obtain electrons from NAD(P)H for oxygen activation and subsequent substrate oxidation. Insufficient CPR electron transfer may lead to reactive oxygen species formation and lower catalytic performance of P450s (Xiao et al., 2019). A promising strategy to optimize the electron flux to P450s is the construction of fusions to CPRs, hence preventing loss of electrons in recombinant microbial cells (Fig. 4A).

CYP450-dependent monooxygenases are important components in Taxol biosynthesis, mediating eight of the proposed nineteen biosynthetic steps. Studies have shown that the activity of Taxol pathway CYP450s is limiting in yeast and *E. coli* (Biggs et al., 2016; Engels et al., 2008). The CYP450 taxadiene 5*α*-hydroxylase catalyzes the first oxygenation step along with double-bond migration in the precursor taxadiene (Fig. 1). Ajikumar et al. (2010) optimized the expression of taxadiene 5*α*-hydroxylase by codon-optimization and engineering of the N-terminal domain via connection of the eight-residue peptide MALLLAVF from bovine 17*α*-hydroxylase to the 24-amino acid truncated transmembrane region. Subsequent fusion of the respective taxadiene 5*α*-hydroxylase construct with the partner CYP450 reductase from the *Taxus* species was found to be highly efficient, resulting in more than 98% taxadiene conversion to taxadien-5*α*-ol and a cyclic ether oxidation product. The introduction of additional copy numbers of the fusion construct reduced the productivity, suggesting that the balance between the upstream and downstream heterologous modules is a critical factor for the effectiveness of the pathway (Ajikumar et al., 2010). Apart from the examples described above, numerous studies have appeared in the literature employing the enzyme fusion approach for isoprenoid production, denoting its high biotechnological applicability (Table 1).

**Table 1:** Summary of studies employing the protein fusion approach for enhanced *in vivo* production of isoprenoids.

Strategy	Host organism	Target compound	Scale/Medium	Titer/Content	Fold-increase	Reference
Fusion variants of *ERG20* and patchoulol synthase gene from *Pogostemon cablin* in an *ERG9*-repressed strain	*S. cerevisiae*	Patchoulol	1.1-liter bioreactor/Galactose-based mineral medium	40.9 ​mg/L	1.2	Albertsen et al. (2011)
Fusion of *BTS1* and *DPP1*	*S. cerevisiae*	(*E*,*E*,*E*)-Geranylgeraniol	Test tube/Yeast malt broth	~1.1 ​mg/L	2.9	Tokuhiro et al. (2009)
Fusion of *SmKSL* and *SmCPS* genes from *Salvia miltiorrhiza*, along with fusion between *BTS1* and *ERG20*	*S. cerevisiae*	Miltiradiene	Flask/YPD broth	3.1 ​mg/L	4.4	Zhou et al. (2012)
Fusion of *ERG20* and bisabolene synthase gene from *Abies grandis*	*S. cerevisiae*	Bisabolene	Flask/Galactose-based minimal broth	~150 ​mg/L	~2	Özaydın et al. (2013)
Fusion of *E. coliispA* and *α*-farnesene synthase gene	*E. coli*	*α*-Farnesene	Flask/Rich (2xYT) medium	86.8 ​mg/L	1.5	Wang et al. (2011)
Construction of a tridomain fusion protein (CrtB, CrtI, CrtY) harboring the *β*-carotene pathway from *Xanthophyllomyces dendrorhous*	*S. cerevisiae*	*β*-Carotene	Flask/YPG broth	~2.7 ​mg/g DCW	2.2	Rabeharindranto et al. (2019)
Fusion of *ERG20* and amorphadiene synthase gene from *Artemisia annua* L.	*S. cerevisiae*	Amorpha-4-11-diene	2-liter bioreactor/Galactose-based mineral medium	25.1 ​mg/L	~2	Baadhe et al. (2013)
Fusion protein of Erg20p(F96C) and *Cc*CLS from *Cistus creticus*	*S. cerevisiae*	Sclareol	Flask/Galactose -raffinose-based selective medium	28.0 ​mg/L	1.8	Ignea et al. (2015b)
Fusion protein of Bts1p and Erg20p(F96C)	*S. cerevisiae*	13*R*-Manoyl oxide	Flask/YPD broth	23.3 ​mg/L	3	Zhang et al. (2019)
Fusion between *ispA* and *Mycobacterium tuberculosisZ*,*E*-FPP synthase gene (Rv1086)	*E. coli*	*Z*,*E*-farnesol	Flask/Modified 2xYT	115.6 ​mg/L	15	Wang et al. (2013)
Fusion of *CrtZ* and *CrtW* genes from from *Brevundimonas* sp. strain SD212	*E. coli*	Astaxanthin	Flask/Luria-Bertani medium	610 μg/g DCW	1.4	Nogueira et al. (2019)

Abbreviations: *BTS1*, gene encoding for geranylgeranyl diphosphate synthase (Bts1p); *DPP1*, gene encoding for diacylglycerol diphosphate phosphatase 1; *ERG9*, gene encoding for squalene synthase; *ERG20*, gene encoding for farnesyl diphosphate synthetase (Erg20p); *ispA*, gene encoding for farnesyl diphosphate synthase; *Sm*KSL, kaurene synthase-like from *Salvia miltiorrhiza*; *Sm*CPS, copalyl diphosphate synthase from *Salvia miltiorrhiza*; CrtB, phytoene synthase; CrtI, phytoene desaturase; CrtY, lycopene cyclase; *Cc*CLS, *Cistus creticus* 8-hydroxy copalyl diphosphate synthase; CrtW, *β*-carotene ketolase; CrtZ, *β*-carotene hydroxylase; DCW, dry cell weight; Yeast extract-Peptone-Dextrose (YPD); Yeast extract-Peptone-Glycerol (YPG); YT, Yeast extract-Tryptone.

In addition to fusion proteins, an alternative strategy to promote co-localization includes the sequential attachment of enzymes to synthetic scaffolds in a programmable and defined spatial order (Fig. 4B) (Pröschel et al., 2015). Synthetic scaffolds constitute multiple interaction domains in which the enzymes of interest are linked together in a modular manner. It is suggested that the probability of intermediate processing is higher in the case of synthetic scaffolds due to the co-localization of enzymes in the cluster providing control over intermediate pathway flux (Castellana et al., 2014). A prominent example of artificial scaffolds is the class of nonimmunoglobulin affinity proteins, namely affibodies, derived from the Fc-binding domain of *Staphylococcus aureus* protein A (Tippmann et al., 2017). Affibodies represent small size and high stability scaffold proteins which combine the favorable molecular recognition properties of antibodies with fast folding kinetics and the possibility of using multispecific constructs (Löfblom et al., 2010). In a study by Tippmann et al. (2017), affinity proteins were employed for the production of farnesene in *S. cerevisiae*. The affibodies Z_Taq_ and Z_IgA_ were tagged to farnesene synthase and FPPS, respectively. In a subsequent step, the respective affibodies were recognized by their anti-idiotypic partners (anti-Z_Taq_ and anti-Z_IgA_) that were linked together to form a binding scaffold. As a result of enzyme scaffolding, the farnesene yield was enhanced up to 135% in fed-batch cultivations. Moreover, it was found that the enzyme:scaffold ratio is a critical factor for improving the yield of farnesene (Tippmann et al., 2017).

Besides affibodies, interaction domains from metazoan signaling proteins constitute another type of attractive artificial scaffolds. In a representative study, the three-enzyme pathway from acetyl-CoA to mevalonate was employed as a model system in *E. coli* (Fig. 1) (Dueber et al., 2009). Accordingly, various synthetic scaffolds were constructed with a different number of repeats of three metazoan signaling proteins, including the GTPase binding domain (GBD), the SH3 domain, and the PSD95/DlgA/Zo-1 (PDZ) domain. The respective domains were recombined in various arrangements to the C-terminus of MVA pathway enzymes using specific interaction ligands. The modularity of the interaction domains was exploited to optimize the stoichiometry of enzymes, resulting in an up to 77-fold improvement in the mevalonate titer in comparison with the non-scaffolded pathway.

The studies mentioned above illustrate that synthetic scaffolds could be successfully implemented in heterologous systems that are limited by multi-step assembly pathways. However, it should be noted that scaffolded enzyme assemblies pose many challenges due to different properties of the individual component enzymes that may result in adverse conformational changes and loss of activity (Jia et al., 2014). To overcome this limitation, Kang et al. (2019) investigated the interaction of short peptide tags, including the RIAD peptide (18 amino acids), which originates from the A kinase-anchoring proteins (AKAPs), and the RIDD peptide (44 amino acids), a dimerization and docking domain of the cyclic AMP-dependent protein kinase (PKA) (Sarma et al., 2010; Kang et al., 2019). It was shown that the RIDD and RIAD peptides possess strong binding affinity at a 2:1 stoichiometry, yielding scaffold-free modular enzyme assemblies. The RIAD–RIDD interaction was employed for the production of carotenoids in both *E. coli* and *S. cerevisiae*, by fusion of the respective tags to the IDI and geranylgeranyl diphosphate synthase (CrtE). The assembly of IDI with the CrtE showed a 5.7-fold increase (276.3 ​mg/L) in the total carotenoid production in *E. coli*. Similarly, the IDI-CrtE complex led to a 58% increase in the lycopene production in *S. cerevisiae*, reaching a final titer of 2.3 ​g/L in fed-batch fermentation (Kang et al., 2019).

### 3.2 Combinatorial biosynthesis of isoprenoids

Combinatorial biosynthesis is a promising approach to exploring artificial metabolic networks for the generation of novel terpene derivatives (Fig. 4C). This strategy takes advantage of the wide array of potential pathways that can be explored by mimicking the modularity of isoprenoid biosynthesis and harnessing the promiscuity of isoprenoid pathway enzymes (Erb et al., 2017). As such, novel enzymatic activities can be obtained through protein engineering, followed by different combinations of enzyme-substrate pairs to unlock the chemical diversity of isoprenoids. For example, Ignea et al. (2015a) exploited the substrate promiscuity of various diterpene synthases leading to an array of diterpenoids, such as miltiradiene, manoyl oxide, and manool in *S. cerevisiae*. By further utilizing these compounds as scaffolds, a range of hydroxylated diterpenes were generated employing a mutant library of promiscuous cytochromes P450. Accordingly, the structural model of *Pinus taeda* abietadiene oxidase was constructed based on the structurally homologous CYP120A1, which revealed several hydrophobic residues in the active site cavity with an effect on product specificity. By narrowing mutagenesis to include only hydrophobic residues of different sizes, the overall pocket volume could be greatly remodeled without compromising its chemical properties. As a result, novel isoprenoids were obtained, including 18-hydroxy miltiradiene, 19-hydroxy miltiradiene, and 19-hydroxy manool for which no dedicated P450 enzymes have been reported so far (Ignea et al., 2015a). In another study, the mangicdiene synthase from *Fusarium graminearum* (*Fg*MS) was found to convert isoprenoid diphosphates of different lengths, such as GPP, FPP, GGPP, and GFPP, to variable isoprenoids in vitro, exhibiting broad substrate promiscuity (Bian et al., 2017). Interestingly, *Fg*MS is a chimeric enzyme possessing both prenyltransferase and terpene synthase activities. Replacement of aspartic acid with alanine at position 510 abolished the prenyl trasferase activity, thus blocking the substrate flux toward GFPP. By the combination of upstream prenyltransferases with the respective *Fg*MS variant, Bian et al. (2017) generated an array of various terpenoids in *E. coli*, many of them possessing new carbon skeletons.

Carotenoids are high-value natural pigments with several applications in the nutraceutical industry due to their colorant and antioxidant properties (Li et al., 2019). Non-natural longer-chain carotenoids accommodating a higher number of conjugated double bonds in microorganisms could be exploited as potential components for novel light-harvesting, photovoltaic, and photonic platforms (Zhuang et al., 2015). ​Furubayashi et al. (2015) ​reported the biosynthesis of various novel C30-C55 carotenoid pigments by combinatorial expression of FPPS and 4,4′-diapophytoene synthase (CrtM) variants (Furubayashi et al., 2015). In a further study, engineering of the C50-phytoene producing CrtM variant shifted the product size specificity to the production of non-natural C60-phytoene along with minor amounts of C65-phytoene, the largest carotenoid that has hitherto been biosynthesized (Li et al., 2019). Likewise, a phytoene desaturase (CrtI) variant was constructed, bearing a single amino acid mutation (i.e., N304P) that is able to convert C50–C55 substrates into non-natural carotenoid pigments, indicating the remarkable plasticity of these enzymes. ​The studies above indicate that protein engineering can supplement the combinatorial biosynthesis strategy, further expanding the biosynthetic repertoire of isoprenoid enzymes.

## 4 Conclusion and future perspectives

The low expression and stability of wild-type isoprenoid enzymes pose many limitations for their successful implementation in heterologous microbial systems. As discussed in this review, various protein engineering strategies have been developed, including enhancement of catalytic activity, varying substrate selectivity, enzyme co-localization, and deregulation of feedback inhibition enabling significant enhancement of isoprenoid titers. Moreover, protein engineering has attracted great interest in the production of new-to-nature isoprenoids, either by constructing variants accommodating a wider range of non-native substrates or by implementing artificial enzyme cascades through combinatorial biosynthesis. These strategies have been efficiently employed in a complementary manner for optimal production of isoprenoids. As an example, fusion of neryl diphosphate synthase (*Sl*NPPS1) to a dominant-negative Erg20p variant that inhibits the FPP synthesis step (i.e., Erg20p(N127W)–*Sl*NPPS1) resulted in an up to 2-fold production of neryl diphosphate (NPP), the cis-isomer of GPP. Subsequently, with the aim to increase NPP specificity, the canonical monoterpene synthases *Citrus limon* (+)-*S*-limonene synthase (*Cl*LimS) and *Salvia pomifera* sabinene synthase (*Sp*SabS) were engineered through rational design leading to 134.8 (4.8-fold increase) and 72.4 ​mg/L (4.1-fold increase) of limonene and sabinene in *S. cerevisiae*, respectively (Ignea et al., 2019). However, a significant limitation comes from the fact that heterologous proteins in multistep metabolic pathways are often constrained by flux imbalances of the host. To this end, once optimized variants of pathway isoprenoid enzymes have been constructed, further metabolic engineering could be applied to boost exploitation of the protein engineering toolkit and enable high production of isoprenoids. This is likewise illustrated in the aforementioned study, in which the promoter of *ERG20* was replaced by an ergosterol-responsive element in order to minimize flux to FPP synthesis when adequate levels of ergosterol were synthesized (Ignea et al., 2019).

The biosynthetic repertoire of isoprenoid enzymes that have hitherto been identified only covers a small portion of the isoprenoids found in nature (Bian et al., 2017). One of the most prominent examples is the anticancer drug Taxol. Even though Taxol (paclitaxel) was isolated in the late 1960s from the Pacific yew (*Taxus brevifolia*), several CYPs involved in its biosynthetic pathway remain uncharacterized, limiting its biotechnological production (Ajikumar et al., 2010). The high interest in the sustainable production of complex value-added isoprenoids is predicted to accelerate the reconstitution of the complete set of enzymes involved in native biosynthetic pathways. Moreover, advanced techniques in genome mining, transcriptomics, and bioinformatics will enhance the identification of new enzymes in isoprenoid biosynthesis (Xiao et al., 2019). The growing number of isoprenoid enzymes is expected to provide a wealth of structural information, especially in the substrate-bound state, that could be used as the basis for rational protein engineering. Nonetheless, the complication in determining the functional and structural role of various residues that are involved in the catalytic activity of different enzymes represents a major limiting factor for the application of rational protein engineering systems in isoprenoid biosynthesis (Renault et al., 2014; Janocha et al., 2015).

Directed evolution is a valuable strategy when structural information regarding the catalytic activity or a specific property of an enzyme is not available. However, in most cases, a large number of mutants need to be screened, which is labor-intensive and is limited by the availability of an efficient screening method (Lauchli et al., 2013; Yoshikuni et al., 2006). In this regard, ultra-high-throughput technologies, such as droplet- and microchamber-based platforms are emerging workflows enabling higher automation and exploration of tens of millions of variants during a library screen (Gielen et al., 2016; Longwell et al., 2017). In addition, mutagenic studies on terpene synthases often rely on tedious GC–MS and LC-MS analyses (Lauchli et al., 2013). To overcome this challenge, visual high-throughput detection assays have been developed, facilitating the connection between color formation and enzyme activity of terpene synthases (Furubayashi et al., 2014). These methods take advantage of the fact that isoprenoid enzymes utilize the same isoprenyl diphosphate substrates, such as FPP and GGPP. Accordingly, co-expression of terpene synthase active variants in carotenoid-producing strains results in decreased availability of building blocks for carotenoid biosynthesis, and thus in lower pigmentation level in the host cells. As such, this method does not only differentiate between active and inactive terpene synthases but also between variants with various levels of activity by assessing the color intensity of the respective colonies. Another advantage of this assay is that it is applicable to other important classes of enzymes, such as prenyltransferases, which are involved in terpene quinone and meroterpene biosynthesis (Furubayashi et al., 2014; Klein-Marcuschamer et al., 2007).

Rational design using computational methods has proven to be another very promising strategy to avoid lengthy screening processes. The development of sophisticated computational methods and algorithms could be efficient in predicting synergistic effects of various mutations, thus reducing the library size by limiting mutations to only those that are predicted to be beneficial (Steiner and Schwab, 2012). Apart from introducing new activities to enzymes through rational engineering or directed evolution, another promising strategy is the design of *de novo* artificial enzymes based on computational design methods (e.g., RosettaDesign). In the “inside-out” approach, quantum calculations of transition states provide fundamental insights into theoretical active sites (theozymes) with catalytic functionality (Kiss et al., 2013). The active sites are subsequently positioned to selected inert protein scaffolds that support their three-dimensional side-chain arrangement and functionality. The *de novo* design approach could provide valuable starting points for biocatalysts with higher stability and improved expression profiles or catalytic rates compared to natural enzymes (Steiner and Schwab, 2012). The construction of *de novo* enzymes is still in its infancy, currently limited to simple catalytic reactions only. However, the impact of computational methods in enzyme engineering is expected to grow rapidly in the coming years.

The protein engineering strategies described in this review have been inspired by the intrinsic promiscuity of individual enzymes as well as by natural multienzyme systems (e.g., cellulosome) that have been generated during billions of years of evolution for optimal activity of pathway enzymes. It is envisaged that a more detailed understanding of the functional role of isoprenoid enzymes in their natural environment and the protein-protein or protein-substrate interaction networks thereof will provide us with new insights and exciting opportunities for the development of novel engineering strategies to boost the microbial production of isoprenoids.

## Declaration of competing interest

The authors declare that they have no known competing financial interests or personal relationships that could have appeared to influence the work reported in this paper.
